# Training, Experiences and Factors Contributing to Learning Variability in Podiatry Residency and Fellowship Programs: A Systematic Review

**DOI:** 10.3390/healthcare14091165

**Published:** 2026-04-27

**Authors:** José Manuel Cuevas-Sánchez, Sergio Barrientos-Trigo, José Algaba-del-Castillo, Manuel Coheña-Jimenez

**Affiliations:** 1 Departamento de Podología, Facultad de Enfermería, Fisioterapia y Podología, Universidad de Sevilla, 41009 Sevilla, Spain; jmcusan@hotmail.com (J.M.C.-S.); algaba@us.es (J.A.-d.-C.); 2 Departamento de Enfermería, Facultad de Enfermería, Fisioterapia y Podología, Universidad de Sevilla, 41004 Sevilla, Spain; sbarrientos@us.es; 3 Research Group PAIDI-CTS 1141 “Clinical Research Applied to Care and New Care Paradigms (ICCAPA)”, Andalusian Public Health Service, 41071 Sevilla, Spain; 4 Research Group PAIDI-CTS 589: Advances in Podiatric Surgery, Andalusian Public Health Service, 41071 Sevilla, Spain

**Keywords:** podiatry, residency, fellowship, surgical training, clinical competencies

## Abstract

**Background/Objectives:** Postgraduate training is an essential component for the development of professional skills in health sciences. Our research question was: How does the implementation of structured residency and fellowship programs in podiatry and foot and ankle surgery impact the acquisition of clinical and surgical skills, academic productivity, interprofessional integration, leadership development, and resident well-being compared to less structured or traditional programs? **Methods**: We conducted a systematic review of published research, between September and November 2025, selecting observational studies that evaluated structured training programs compared to traditional approaches. The studies included reported residency or fellowship programs in podiatry and foot and ankle surgery that described clinical, surgical or academic experiences, together with the factors that influence learning variability. **Results**: Eleven cross-sectional studies were included. Program structure, mentorship, clinical exposure, availability of educational resources, and individual motivation are determining factors in the variability of skills acquisition. Structured programs were associated with better academic and clinical performance, greater technical confidence, and professional leadership development. However, substantial heterogeneity was observed among programs, particularly regarding access to resources, which contributed to differences in the ultimate preparation of residents and fellows. The Newcastle–Ottawa Scale adapted assessed methodological quality, showing a low-to-moderate risk of bias. **Conclusions**: The literature suggests that although the programs generally achieve basic training objectives, the standardization and implementation of structured educational strategies could optimize skills acquisition and reduce variability across programs. Furthermore, multicenter research incorporating objective outcome measures would facilitate the development of internationally applicable standards for evaluation in health education.

## 1. Introduction

Postgraduate training in podiatry, specifically within foot and ankle surgery, is a critical determinant of clinical and surgical excellence in musculoskeletal care. Residency and fellowship programs provide structured clinical, surgical and academic environments, designed to foster advanced technical proficiency, research competencies and leadership in multidisciplinary settings [1,2,3]. Despite the progressive standardization of these curricula, current literature suggests significant variability in competency acquisition, potentially mediated by diverse institutional and systematic factors [4,5,6].

Recent analyses have focused on the academic productivity of podiatric surgeons affiliated with such programs. Participation in structured training programs correlates with a significant increase in enhanced scientific output and the development of academic leadership [1]. For instance, Chu et al. demonstrated that cadaveric laboratories significantly improve technical skill acquisition during residence [2], while King et al. (2024) described a synergistic model at Kaiser Permanente that integrates clinical rotations with research and committee involvement [3]. Consequently, the architecture of these programs directly influences residents’ perceptions of training adequacy and overall well-being [4], with fellowships serving as a strategic adjunct to post-resident clinical maturation [5].

Recently, Rushing et al. reported that advanced training enhances job competitiveness and the management of complex cases, justifying the investment in professional development [6,7]. Roukis (2024) further emphasized that structured fellowships facilitate subspecialization and the cultivation of clinical leaders in high-complexity centers [8].

However, factors such as accreditation disparities [9]. excessive workload, inadequate supervision, and academic pressure negatively impact both resident satisfaction and clinical performance [10,11]. While interdisciplinary perspectives from broader medical education offer valuable benchmarks [12,13,14,15], a specific standardized framework for podiatric surgery is imperative to ensure patient safety [16,17]. Moreover, incorporating ethical training is fundamental in postgraduate education to strengthen decision-making [18]. Psychological stress in orthopedic and trauma residents—such as burnout from imbalanced workloads—remain fundamental to modern postgraduate education [19].

Effective skill acquisition relies on evidence-based planning, clinical demand alignment, and robust supervision [20,21,22,23,24]. Such specialized training consolidates both technical and leadership domains [25,26], yet observed variability among orthopedic surgery residents underscores the need for standardization and structured supervision to enhance clinical competence [27,28,29]. Additionally, active teaching methodologies and equitable supervision are essential to mitigate gender bias and enhance clinical decision-making in surgical training [30,31,32,33]. Overall, postgraduate training in podiatry through residency and fellowship programs is multifactorial and complex. Given the multifactorial complexity of podiatric postgraduate education, optimizing the training of foot and ankle surgeons requires a balance between standardization and local adaptability.

This systematic review aims to synthesize existing evidence to identify factors that contribute to learning variability and provide recommendations for program enhancement. The primary research question is: What training characteristics, educational experiences, and contextual factors are associated with variability in clinical and surgical skill acquisition, academic productivity, interprofessional integration, leadership development, and resident well-being in podiatry and foot and ankle surgery residency and fellowship programs? The main objective of this systematic review was to analyze the training, experiences and factors that contribute to variability in learning in podiatry residency and fellowship programs.

## 2. Materials and Methods

This systematic review was conducted using the PICO question strategy: patients (subjects), intervention, comparison, and outcomes. The PRISMA checklist was used (Supplementary Materials) [34]. This review is registered in PROSPERO: CRD42024604497. The planned research question is as follows: What training characteristics, educational experiences, and contextual factors are associated with variability in clinical and surgical skill acquisition, academic productivity, interprofessional integration, leadership development, and resident well-being in podiatry and foot and ankle surgery residency and fellowship programs?

In our study, the population of interest consists of residents and fellows participating in training programs in podiatry and foot and ankle surgery (P). The intervention corresponds to Structured residency and fellowship programs with clinical supervision, diversified rotations, cadaver laboratories, active teaching methods, and mentorship (I). Rather than establishing a direct comparison between structured and less structured or traditional programs, the comparison component focused on variation across training settings, program characteristics, and educational experiences when these were reported in the included studies (C). The results cover multiple dimensions of professional development, including the acquisition of clinical and surgical skills, academic productivity, interprofessional integration, leadership skills development, and the overall well-being of residents during their training (O).

### 2.1. Search Strategy

When carrying out the search strategy to locate all the information available in the various Health Sciences databases, the focus was placed on key concepts related to participants, interventions and study design, with special attention to ensuring that these terms are found in the title or abstract of the documents. The systematic review prioritized observational studies and clinical trials that met the predefined selection criteria. For the current systematic review, we specifically examined training, experiences and factors contributing to variability in learning within residency and fellowship programs in podiatry and foot and ankle surgery. Using this approach, the literature search yielded 3649 potentially relevant publications.

### 2.2. Databases and Literature Search

The literature search was conducted between September and November 2025, consulting databases such as MEDLINE (US National Library of Medicine, Bethesda MD), PubMed, Scopus TM (Elsevier, Amsterdam, The Netherlands), Web of Science, Embase (Elsevier, Amsterdam, The Netherlands), The Cochrane Library, and CINAHL. Combinations of MeSH and DeCS terms were used, including “Podiatry”, “Residency”, “Fellowship”, “Surgical training” and “Clinical competencies” (a detailed description is included in Table 1).

### 2.3. Inclusion and Exclusion Criteria, Study Selection

Original studies with a cross-sectional methodological design were included, as well as reports from residency or fellowship programs in podiatry and foot and ankle surgery that described clinical, surgical or academic experiences, together with the factors that influence learning variability. In addition, studies that evaluated academic productivity, interprofessional integration or the well-being of residents were considered.

Conversely, all literature related to medical or surgical specialties other than podiatry, documents in languages other than English or Spanish, and all grey literature lacking scientific rigor, including conference proceedings, books, isolated clinical cases, editorials, and letters to the editor, were excluded. Narrative reviews, consensus statements, and studies focused exclusively on educational tools not directly linked to residency or fellowship programs, such as simulations unrelated to structured programs, were also excluded.

All references obtained were managed using the Zotero bibliographic manager, eliminating duplicates and reducing the initial total of 172 articles to 34. The selection was carried out in two phases. First, two independent reviewers (A1 and A2) screened titles and abstracts, evaluating the studies according to the inclusion and exclusion criteria.

**Figure 1:**
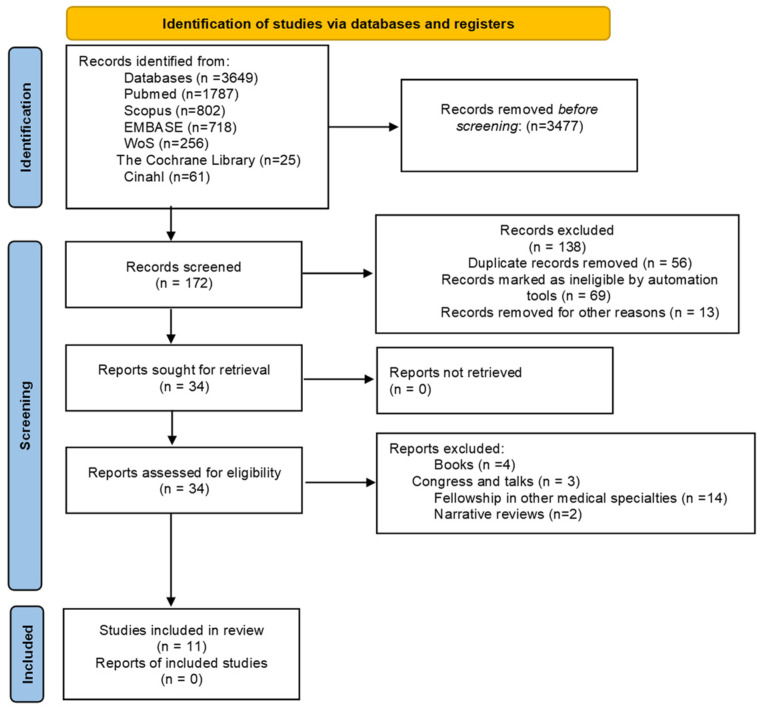
PRISMA Flow-chart of articles included in the systematic review.

Next, a full text review was conducted, selecting those studies that fully met these criteria, resulting in a total of 34 articles. From this set, observational studies and clinical trials that provided direct evidence on training and variability in podiatry programs were prioritized, resulting in the inclusion of 11 studies. The procedure carried out for data selection is represented by a flowchart or flow diagram based on the principles of the PRISMA statement (Figure 1). A data extraction table was developed to systematically record relevant information from the included studies: author, year, study design, journal, objectives, sample population, duration, teaching methods, clinical rotations, academic and clinical outcomes, learning variability factors, conclusions, level of evidence, and grade of recommendation. The information was extracted independently by both reviewers and then cross-checked to ensure accuracy and consistency.

### 2.4. Risk of Bias Analysis

The risk of bias of the studies included in this systematic review was assessed using the adapted version of the Newcastle–Ottawa Scale for cross-sectional studies (NOS-xs) [35]. Given that all the studies included have a cross-sectional observational design, a single tool specific to this type of study was used, allowing for a homogeneous and methodologically consistent assessment. The NOS-xs scale assesses three domains: sample selection (0–2 points), exposure/outcome (0–4 points) and control of confounding factors (0–3 points), with a maximum total score of nine stars. Based on the total score, studies were classified as having low (7–9 stars), moderate (4–6 stars) or high (0–3 stars) risk of bias.

Of the eleven cross-sectional studies included, six were classified as having low risk of bias. These studies showed adequate sample representativeness, valid and reliable measurement of outcomes, and appropriate control of confounding factors. The study by Silvestre et al. [9] achieved nine stars, reflecting high methodological quality in the three domains evaluated. Four studies presented a moderate risk of bias (6 stars), mainly due to limitations in the exposure/outcome domain or partial adjustment for confounding factors. Finally, one study was classified as having a high risk of bias (2 stars), attributable to limited sample selection, unobjective measurements, and absence of control for confounding factors.

The main methodological limitation common to the included cross-sectional studies is the single point measurement of exposures and outcomes, without longitudinal follow-up, which prevents the establishment of causal relationships. Nevertheless, most studies are of acceptable to high methodological quality, supporting a cautious but reliable interpretation of the findings.

Details of evaluation are included in the following tables, evaluation of methodological quality (Table 2), assessment of the risk of bias (Table 3), and chromatic visual representation of the assessment of the risk of bias for cross-sectional observational studies using NOS-xs (Table 4).

## 3. Results

### 3.1. Summary of Results

The characteristics of the studies and the most relevant findings are presented in Table 5, which details the interventions, instruments used, results obtained, and conclusions of each study. This presentation allows for comparison of the different residency and fellowship programs, as well as identification of the factors that contribute to learning variability.

### 3.2. General Characteristics of the Included Studies

Eleven cross-sectional observational studies were included, with a combined total of 903 participants, which analyzed different aspects of training in podiatry and podiatric surgery residency and fellowship programs. Most used descriptive designs or cross-sectional surveys aimed at characterizing the experience of residents, quantifying their academic productivity, and exploring the factors that influence the acquisition of clinical and surgical skills. The publications cover the period from 2013 to 2025 and come mainly from the United States, supplemented by studies from Turkey that provide an international perspective on specialized training.

The populations included residents in podiatric surgery, foot and ankle fellows, and, in some cases, residents in orthopedics and traumatology with comparable training. The sample size varied widely, from single-center studies with fewer than 6 participants to multicenter studies with more than 159 subjects. Most studies employed a cross-sectional design, although some included cohort follow-up or evaluated specific educational interventions, such as cadaver laboratories or external rotations, typically lasting approximately one academic year.

### 3.3. Clinical Experience and Surgical Exposure

Studies agree that direct clinical exposure and surgical practice opportunities are the most important pillars of skill acquisition during residency. Programs with a more diversified rotational structure provide greater access to complex clinical cases, thereby promoting autonomous decision-making [3]. Marked heterogeneity was also observed among fellowship programs [5]. However, exposure to external environments improves adaptability, clinical judgement, and problem-solving, reinforcing the skills needed for unstructured or more complex clinical scenarios [28].

### 3.4. Academic Productivity and Professional Development

Academic productivity (publications, presentations, participation in research projects) showed considerable variability between programs. Institutional support and mentorship directly influence academic performance [1]. Conversely, structural limitations, such as high caseloads, limited availability of academic mentors, and lack of protected time, restrict residents’ research capacity. Complementarily, fellowship programs tend to enhance academic productivity through more structured mentorship, access to multicenter projects, and greater professional interaction [7]. If this is accompanied by additional scholarships, the academic, professional, and leadership benefits compensate for the investment, especially in the long term [6]. Although a formal meta-analysis was not possible, several studies provided descriptive quantitative metrics on the academic productivity of residents and fellows in podiatry and foot and ankle surgery programs.

### 3.5. Factors Contributing to Learning Variability

The studies identified multiple determinants contributing to the heterogeneity observed in clinical and academic training. Program structure emerged as the most influential factor [3,28]. On the other hand, programs with accessible tutors and clearly defined support strategies encouraged greater academic productivity and professional satisfaction [5,7]. In addition, access to educational resources had a positive association with surgical preparedness [3] and high demand for care with the limitation of the acquisition of advanced skills in research [11]. Finally, personal factors such as intrinsic motivation, interest in research, and individual proactivity were related to better academic and professional outcomes [1,6].

### 3.6. Evaluation of Educational Outcomes and Acquired Skills

Overall, residency and fellowship programs allowed for the development of clinical, surgical, and academic competencies. Key skills acquired included technical skills for foot and ankle procedures, research and academic competencies, such as scientific writing and conference participation, independent decision-making, and preparation for leadership roles in highly specialized clinical settings. Exposure to external rotations and complex cases promoted advanced clinical reasoning [3,28], and programs with robust mentorship and networking opportunities demonstrated a greater impact on professional development [6,32]. Despite the positive aspects, the review revealed several structural limitations that affect the uniformity and effectiveness of training. These studies demonstrate that the evidence is limited, as they do not evaluate the actual performance measures of graduates from these programs.

### 3.7. Methodological Quality and Risk of Bias

The methodological quality of the studies was assessed using the Newcastle–Ottawa Scale (NOS) for cross-sectional studies [35]. The adapted version of the scale, known as NOS-xs, retains the original three-domain structure. The sample selection domain evaluates the representativeness of the population, the sample size, and the adequacy of the inclusion and exclusion criteria. The exposure or outcome domain analyses the validity and reliability of the measurements and the clarity of variable definitions. Finally, the confounding factors control domain assesses the identification and adjustment of possible confounding factors through the study design or statistical analysis.

This scale was systematically applied to the eleven cross-sectional studies included, enabling a consistent and appropriate assessment of the risk of bias in accordance with the characteristics of this study design. A common methodological limitation among these studies is the cross-sectional measurement of exposure and outcome, without temporal follow-up, which prevents the establishment of causal relationships. Nevertheless, most studies demonstrated acceptable-to-high methodological quality, supporting a cautious but reliable interpretation of the findings.

### 3.8. Level of Evidence and Grade of Recommendation

Given that all the studies included in the review were observational cross-sectional studies, the level of evidence is classified as 2b according to the Oxford Centre for Evidence-Based Medicine [36]. Based on this framework, the body of evidence obtained in the review receives a type B recommendation, indicating that, although the studies lack an experimental design, their findings are consistent and provide useful and reliable conclusions for optimizing podiatry training and residency and fellowship programs [37].

## 4. Discussion

This systematic review provided a comprehensive analysis of the training, experiences, and factors contributing to learning variability in podiatry and podiatric surgery residency and fellowship programs. The eleven observational studies included allowed us to identify consistent patterns, as well as factors determining heterogeneity in the acquisition of clinical, surgical and academic skills. The reviewed literature suggests that, although the programs meet the fundamental training objectives, there is still considerable variability between them, influenced by structural factors, available resources, mentorship and the individual motivation of residents.

One of the most significant findings of the reviewed studies was the importance of direct clinical experience and surgical exposure in competency development. Programs incorporating experimental laboratories were associated with improved performance in complex and unforeseen surgical procedures, particularly when complemented by prior experience gained through external rotations [3,28]. These findings support the notion that the diversity and complexity of clinical experience are essential determinants for the training of competent and confident professionals in podiatric surgery.

Academic productivity emerged as a critical component of advanced training, although substantial variability was observed across programs. Residents and fellows with access to structured mentorship, multicenter projects, and protected time for research demonstrated higher levels of publications and presentations, while those in programs with less academic support faced significant limitations [1,7]. Moreover, investment in advanced training is associated with the development of sustained competencies and long-term professional leadership capacity [6].

The heterogeneity of programs, identified as a recurring factor, manifested itself in multiple dimensions. Such variability results in pronounced discrepancies in the clinical proficiency of residents and fellows, fostering disparities in professional self-efficacy and performance during the post-residency transition [5]. This finding aligns with extant medical education literature, which posits that a lack of standardized frameworks in specialized training may induce disparities in competency acquisition and, consequently, compromise the caliber of professional practice.

A high clinical workload was associated with reduced time for deep learning and participation in academic activities. The lack of protected time can negatively impact academic productivity, the acquisition of advanced technical skills, and professional satisfaction, creating inequalities among program participants [11]. Additionally, variability observed in fellowship programs was influenced by the clarity of program objectives and structure. Programs with well-defined educational goals and explicit expectations produced fellows with more uniform clinical and academic competencies [5].

Another key factor identified in the studies is mentorship and supervision. Structured mentorship not only guides residents in their clinical development but also supports academic and professional planning, fostering the professional’s comprehensive growth [5,7]. In contrast, programs with limited mentorship reported lower levels of academic development and less confidence in clinical decision-making, underscoring the importance of integrating systematic mentorship strategies into program planning. Educational and technological resources emerged as elements that enhance learning and reduce heterogeneity in training [3]. These findings suggest that investment in educational infrastructure and simulation technologies is a strategic component in improving the quality of training in podiatry and podiatric surgery.

Considering all these findings, podiatry residency and fellowship programs meet the basic objectives of clinical and surgical training, but structural and resource heterogeneity leads to substantial differences in the skills acquired. Technical skills, clinical judgement, and independent decision-making abilities are particularly enhanced by exposure to diverse rotations and practical experience in laboratories and simulation-based training. Similarly, academic productivity is influenced by the availability of mentorship, protected research time, and access to professional networking opportunities. Evidence indicates that the combination of these factors produces better-prepared professionals with greater academic and clinical leadership skills.

These findings have important implications for the practice and design of podiatry education programs. The evidence suggests that it is necessary to standardize clinical rotations, guarantee external experiences and provide protected time for research, ensuring that all residents have access to equivalent learning opportunities. In addition, structured mentorship and clearly defined educational objectives are essential to reduce variability in acquired competencies and promote the comprehensive development of professionals. Likewise, providing protected time for research and developing academic collaboration networks could reduce the observed variability and improve scientific productivity.

Future research should prioritize prospective, multicenter studies incorporating objective outcome measures to enable a more robust evaluation of the impact of specific educational interventions and to generate higher-quality evidence-based recommendations.

Despite these findings, this review also identified significant limitations in the current literature. Although this systematic review provides a comprehensive analysis of podiatric surgery training, it is important to consider certain limitations inherent in the available evidence. First, most of the included studies are cross-sectional observational studies, which limits the ability to establish causal relationships between educational interventions and learning outcomes. Additionally, methodological heterogeneity across studies, including variations in study design, sample size, and competency assessment, hinders direct comparison of results and restricts the generalizability of the findings. The specific literature on postgraduate podiatry training, especially in residencies and fellowships, is still scarce and fragmented.

Furthermore, a geographical bias is evident, as most studies originate from the United States, with only a limited number representing international experiences, such as those from Turkey. This imbalance may limit the applicability of the findings to other educational contexts with different residency and fellowship program structures. Additionally, the lack of longitudinal follow-up precludes the assessment of the sustained impact of interventions on clinical competence, academic productivity, and long-term professional development.

The narrative quantitative analysis conducted in this review enables the identification of patterns and trends in the academic productivity and professional development of residents and fellows. Structured programs appear to promote the generation of publications, participation in multicenter projects, and the development of academic competencies. However, the inability to perform a formal meta-analysis stems from the lack of homogeneous and comparable data across studies, the heterogeneity of reported outcomes, and the predominance of cross-sectional observational designs, which limit the statistical pooling of effects. This methodological limitation underscores the need for future studies that collect standardized and comparable quantitative data, thereby enabling the conduct of formal meta-analysis and providing more precise estimates of the impact of structured programs on podiatric and surgical training. A significant limitation of this review stems from the need to establish similarities between podiatry and orthopedics or traumatology studies, taking into account the established competency levels in Spain.

Furthermore, future research should explore the interaction among structural, technological, and motivational factors, identifying strategies that maximize competency acquisition and reduce heterogeneity among programs. It would also be relevant to include comparative evaluations across different geographic contexts and educational systems to generate globally applicable evidence. Finally, the incorporation of objective indicators of clinical and academic performance, along with validated instruments to assess well-being and professional satisfaction, would contribute to strengthening the evidence base and guiding the continuous improvement of podiatry training programs.

## 5. Conclusions

In general, the literature suggests that the integration of structured clinical training, adequate educational resources, effective mentorship, and academic opportunities contributes to reducing heterogeneity among programs and optimizes the acquisition of clinical and academic competencies. This review has shown the existing variability in the structure, resources and mentoring in graduate learning. The reviewed literature indicates that standardizing clinical rotations, implementing laboratories and simulators, providing protected research time, and offering structured mentorship are essential strategies for improving uniformity in training and preparing residents and fellows for independent professional practice and academic leadership. Additionally, the findings underscore the need for prospective, multicenter studies that evaluate the long-term impact of these interventions, thereby generating robust evidence to inform the optimization of educational programs in podiatric surgery. The present conclusions are strictly aligned with the study’s limitations and reflect the cross-sectional framework of the evidence base. Therefore, the generalizability of these results should be considered within this specific context.

## Figures and Tables

**Table 1:** Search strategy in different specialized databases in Health Sciences.

Database	Search Strategy	Selected Results
Pubmed	(“Internship and Residency”[MeSH] OR “residency program” OR fellowship) AND (training OR experience OR learning OR “clinical skills” OR “clinimetric properties” OR clinimetric) AND (“podiatry surgery” OR “Podiatry”[MeSH] OR “Traumatology”[MeSH] OR “Orthopedic Procedures”[MeSH])	55
Scopus	“residency program” OR fellowship) AND (training OR experience OR learning OR “clinical skills” OR “clinimetric properties” OR clinimetric) AND (“podiatry surgery” OR podiatry OR traumatology OR “orthopedic surgery”)	43
Embase	(‘residency’/exp OR ‘residency program’:ti,ab OR fellowship:ti,ab) AND (training:ti,ab OR experience:ti,ab OR learning:ti,ab OR ‘clinical skills’:ti,ab OR ‘clinimetric properties’:ti,ab OR clinimetric:ti,ab) AND (‘podiatry surgery’:ti,ab OR ‘podiatry’/exp OR ‘traumatology’/exp OR ‘orthopedic procedures’/exp)	38
Web of Science	(“residency program” OR fellowship) AND (training OR experience OR learning OR “clinical skills” OR “clinimetric properties” OR clinimetric) AND (“podiatry surgery” OR podiatry OR traumatology OR “orthopedic surgery”)	29
The Cochrane Library	(“residency program” OR fellowship) AND (training OR experience OR learning OR “clinical skills” OR “clinimetric properties” OR clinimetric) AND (“podiatry surgery” OR podiatry OR traumatology OR “orthopedic surgery”)	3
CINAHL	(MH “Internship and Residency” OR “residency program” OR fellowship) AND (training OR experience OR learning OR “clinical skills” OR “clinimetric properties” OR clinimetric) AND (“podiatry surgery” OR (MH “Podiatry”) OR (MH “Traumatology”) OR (MH “Orthopedic Procedures”))	4

**Table 2:** Evaluation of methodological quality and risk of bias using the Newcastle–Ottawa-xs scale for cross-sectional observational studies.

Study ID	Items Scale			Score NOS-xs (0–9)	Category RoB
Casciato et al. (2021) [1]	2	3	2	7	Low
King et al. (2024) [3]	2	3	2	7	Low
Shofler et al. (2015) [4]	2	2	2	6	Moderate
McAlister et al. (2013) [5]	2	2	2	6	Moderate
Rushing et al. (2021) [6]	2	3	2	7	Low
Shofler et al. (2020) [7]	2	3	2	7	Low
Silvestre et al. (2024) [9]	2	4	3	9	Low
Deheer et al. (2022) [10]	2	2	2	6	Moderate
Demirtaş et al. (2020) [11]	2	2	2	6	Moderate
Shofler et al. (2019) [28]	2	3	2	7	Low
Zgonis and Hyer (2022) [32]	1	1	0	2	High

Abbreviations: Scale NOS-xs (0–9).

**Table 3:** Assessment of the risk of bias using the Newcastle–Ottawa-xs scale for cross-sectional observational studies.

Study ID	Selection	Exposition/Results	Confounding Factors
Casciato et al. (2021) [1]	Low	Low	Low
King et al. (2024) [3]	Low	Low	Low
Shofler et al. (2015) [4]	Low	Unknown	Low
McAlister et al. (2013) [5]	Low	Unknown	Low
Rushing et al. (2021) [6]	Low	Low	Low
Shofler et al. (2020) [7]	Low	Low	Low
Silvestre et al. (2024) [9]	Low	Low	Low
Deheer et al. (2022) [10]	Low	Unknown	Low
Demirtaş et al. (2020) [11]	Low	Unknown	Low
Shofler et al. (2019) [28]	Low	Low	Low
Zgonis and Hyer (2022) [32]	Unknown	High	High

**Table 4:** Chromatic visual representation of the assessment of the risk of bias with the Newcastle–Ottawa-xs scale for cross-sectional observational studies.

Study ID	Selection	Exposition/Results	Confounding Factors
Casciato et al. (2021) [1]	🟢	🟢	🟢
King et al. (2024) [3]	🟢	🟢	🟢
Shofler et al. (2015) [4]	🟢	🟡	🟢
McAlister et al. (2013) [5]	🟢	🟡	🟢
Rushing et al. (2021) [6]	🟢	🟢	🟢
Shofler et al. (2020) [7]	🟢	🟢	🟢
Silvestre et al. (2024) [9]	🟢	🟢	🟢
Deheer et al. (2022) [10]	🟢	🟡	🟢
Demirtaş et al. (2020) [11]	🟢	🟡	🟢
Shofler et al. (2019) [28]	🟢	🟢	🟢
Zgonis T & Hyer CF (2022) [32]	🟡	🔴	🔴

Legend: 🟢 Low risk 🟡 Unknown risk 🔴 High risk.

**Table 5:** Results of the included studies.

Author (Year)	Study Design/Country	Study Objective	Population (N)/ Sample/ Sex	Outcomes/Main Results	Conclusions
Casciato et al. (2021) [1]	cross-sectional/EE. UU.	Academic Productivity in Residency and Fellowship Programs.	Podiatry Residents and Fellows, (N = 106; M 83, F 23)	AV: Total publications, first author publications, senior author publications, total citations, h-index, productivity index adjusted for years of practice. DV: Sex, academic rank, years of practice, institutional affiliation. Institutional mentorship and years of practice correlated with greater academic productivity.	Institutional mentorship and years of practice correlated with greater academic productivity. Differences by sex were observed in the number of publications and citations.
King et al. (2024) [3]	cross-sectional/EE. UU.	Surgical Education at Kaiser Permanente.	Podiatry Residents, N = 60 Sex: Not specified	AV: number and type of procedures performed, complexity of cases, autonomy in surgery. Education: structure of rotations, duration, supervision, access to complex cases, participation in conferences and workshops.	Rotational planning optimizes surgical training and the development of clinical autonomy. Diversified rotations improve exposure to complex cases, competence, and autonomy.
Shofler et al. (2015) [4]	cross-sectional/EE. UU.	Training experience in residencies.	Podiatry residents, N = 45 Sex: Not specified	AV: patients seen, exposure to complex cases, procedures assisted and performed, direct supervision. Training variables: development of clinical and surgical skills, satisfaction with hands-on learning.	Diversified clinical training is crucial for strengthening advanced skills. Intensive practical exposure to complex cases improves decision-making and surgical skills.
McAlister et al. (2013) [5]	cross-sectional/EE. UU.	Characteristics of fellowships in podiatry	Fellows, N = 40 Sex: Not specified	SV: Total fellowship duration, number and type of rotations, level of supervision, clinical-research balance, research opportunities, number of procedures performed, access to academic mentorship.	Standardization of rotations and supervision can improve the clinical and academic competence of fellows. Significant variability in rotations and supervision affects final preparation.
Rushing et al. (2021) [6]	cross-sectional/EE. UU.	Financial implications of undertaking a fellowship	Fellows, N = 35 Sex: Not specified	EV: Annual clinical income, net present value (NPV), return on investment, tuition costs, time spent in fellowship, paid research opportunities. AV: career opportunities, publications, and academic participation. Fellowship increases long-term NPV (approximately $1.2 million more), and benefits exceed initial investment.	Fellowship is a strategic investment; cost–benefit analysis should include future income, academic opportunities, and professional development.
Shofler et al. (2020) [7]	cross-sectional/EE. UU.	Academic productivity in fellowships	Fellows, N = 49; F 24, M 25	AV: Number of publications, principal/senior authorship, conference participation, multicenter projects, collaborations, mentorship received and evaluated. SV: Availability of mentorship, access to research resources. Mentorship and multicenter projects increase academic productivity.	Fellowship programs enhance academic outcomes. Programs that promote structured mentorship and multicenter opportunities increase visibility and scientific output.
Silvestre et al. (2024) [9]	cross-sectional/, EE. UU.	ACGME accreditation in orthopedics	Orthopedic foot and ankle fellows, N = 60 Sex: Not specified	AV: compliance with ACGME standards, methodological quality, documentation of clinical competencies, risk of bias, monitoring of learning outcomes, and evaluation of clinical and academic competencies.	Low risk of bias, good methodological quality. Formal accreditation ensures educational standards and effectively prepares fellows. Accredited programs demonstrate robust methodology and a low risk of bias.
Deheer et al. (2022) [10]	cross-sectional/EE. UU.	Resident well-being	Podiatry residents, N = 80 (N = 121; M 70, F 51)	Well-being variables: levels of perceived stress, burnout, job satisfaction, clinical workload, available institutional resources for support, and stress management strategies.	Supportive interventions needed for well-being. Implementing wellness and clinical workload management programs is critical to optimizing performance and preventing burnout.
Demirtaş et al. (2020) [11]	cross-sectional/, Turquía	Academic productivity and obstacles	Orthopedic residents, N = 116 (M 114 male, F 2)	AV: Number of publications, participation in research, access to mentorship, supervision, and institutional support. SV: clinical workload, time constraints, availability of educational resources, and research opportunities. Structural constraints and clinical workload significantly reduce academic productivity. Structural limitations reduce research.	Improving institutional structure and strengthening mentorship increases productivity and comprehensive development of residents.
Shofler et al. (2019) [28]	cross-sectional/EE. UU.	Off-duty rotations	Podiatry residents, N = 45 Sex: Not specified	AV: Number and type of external rotations, exposure to complex scenarios, clinical autonomy, adaptation to new environments, problem-solving, clinical judgment.	External rotations strengthen advanced skills. Integrating structured external rotations improves advanced skills and preparation for complex clinical scenarios.
Zgonis and Hyer (2022) [32]	cross-sectional/EE. UU.	Fellowship legacy	Fellows, N = 35 Sex: Not specified	AV: Final clinical competence, surgical skills, independence, bias control in assessment, variability between programs, access to mentorship, and academic follow-up. High variability in final preparation; limited methodological assessment generates a critical risk of bias.	Lack of bias control and structure generates critical risk. It is necessary to standardize metrics and methodologies to effectively evaluate the impact of fellowships and ensure uniform quality in final competencies.

Abbreviations: M: Male; F: Female; AV: Academic variables; DV: Demographic variables; SV: Structural variables; EV: Economic variables.

## Data Availability

No new data were created or analyzed in this study. Data sharing is not applicable to this article.

## Supplementary Materials

The following supporting information can be downloaded at: https://www.mdpi.com/article/10.3390/healthcare14091165/s1, PRSIMA 2020 Checklist.

## References

[1] Casciato D.J., Cravey K.S., Barron I.M. (2021). Scholarly productivity among academic foot and ankle surgeons affiliated with US podiatric medicine and surgery residency and fellowship training programs. J. Foot Ankle Surg..

[2] Chu A.K., Law R.W., Greschner J.M., Hyer C.F. (2020). Effectiveness of the cadaver lab in podiatric surgery residency programs. J. Foot Ankle Surg..

[3] King C.M., Neagu C., Williams G. (2024). Shaping the next generation of foot and ankle surgeons: Podiatric surgical residency education at Kaiser Permanente Northern California. Clin. Podiatr. Med. Surg..

[4] Shofler D., Chuang T., Argade N. (2015). The residency training experience in podiatric medicine and surgery. J. Foot Ankle Surg..

[5] McAlister J.E., Hyer C.F., Miller J.M., Rush S., Saxena A., Steinberg J.S., Weil L. (2013). DPM Foot and ankle fellowships. Foot Ankle Spec..

[6] Rushing C.J., Ansert E., Hyer C. (2021). The financial implications of podiatric foot and ankle fellowship: Is another year worth it?. J. Foot Ankle Surg..

[7] Shofler D., To A., Cramer K., Batra S. (2020). Fellowships in podiatric medicine. J. Foot Ankle Surg..

[8] Roukis T.S. (2024). Podiatric foot & ankle surgery fellowships. Foot Ankle Surg..

[9] Silvestre J., Nelson C.L., Thompson T.L., Kang J.D. (2024). Trends in ACGME accreditation of orthopedic surgery fellowship training. Orthopedics.

[10] Deheer P.A., Wolfe W., Nichols J.A., Badell B.J., Patel N.A. (2022). Podiatric medical resident wellness: A group survey study. J. Am. Podiatr. Med. Assoc..

[11] Demirtaş A., Karadeniz H., Akman Y.E., Duymuş T.M., Çarkcı E., Azboy İ. (2020). Academic productivity and obstacles encountered during residency training: A survey among residents in orthopedics and traumatology programs in Turkey. Acta Orthop. Traumatol. Turc..

[12] Ahmad A.S., Mulligan K.M., Zheng D.X., O’Connell K.A., Gallo-Marin B., Koshelev M.V. (2023). The current state of fellowship leadership in dermatology: Trends in workforce demographics and scholarly productivity. Clin. Exp. Dermatol..

[13] Alsheikh S., AlGhofili H., Altoijry A., AlMuhanna G., Alanezi T., Almogren M., Iqbal K. (2024). An integrated vascular surgery residency program would increase interest among Saudi medical students in a career in vascular surgery. BMC Med. Educ..

[14] Balagtas M., Gocal J., Saraf S.M., Mulcahey M.K. (2025). Orthopaedic research year fellowships: Enhancing medical student productivity and hands-on experience. J. Am. Acad. Orthop. Surg. Glob. Res. Rev..

[15] Brickley S., Barrolle S., Pentland A. (2024). Implementation of a postgraduate dermatology fellowship program for nurse practitioners. J. Am. Assoc. Nurse Pract..

[16] Brown R., Bendall S., Aronow M., Ramasamy A. (2024). Podiatrists who perform surgery: Outcomes, regulation, and expanding scope. Bone Jt. J..

[17] Fox J.M., Wason K., Beers D., Faulds M., Lincoln N., Tomanovich M., Gaden N.W., Komaromy M. (2023). The creation of an addiction nursing fellowship program for registered nurses: A unique approach to enhancing the addiction-treatment workforce. Subst. Abus..

[18] Gitin A., Samia A., Grant-Kels J.M., Saikaly S.K. (2023). Ethics training in dermatology residency programs: A survey of dermatology residency program directors and assistant/associate program directors. Clin. Dermatol..

[19] Gülenç B., Yalçin S., Sürücü S., Mahiroğullari M., Erdil M., Bülbül M. (2019). Orthopedics and traumatology residency-working conditions, training, and psychological stress. Acta Chir. Orthop. Traumatol. Cech..

[20] House T.R., Wightman A., Smith J., Schwarze M., Bradford M.C., Rosenberg A.R. (2023). Palliative care training in pediatric nephrology fellowship: A cross-sectional survey. Kidney360.

[21] Ibrahim H., Harhara T. (2022). Palliative care training: A national study of internal medicine residency program directors in the United Arab Emirates. BMC Palliat. Care.

[22] Kesten K.S., Beebe S.L. (2021). Competency frameworks for nurse practitioner residency and fellowship programs: Comparison, analysis, and recommendations. J. Am. Assoc. Nurse Pract..

[23] Kidd V.D., Douglas G. (2024). Perceptions of nurse practitioners and physician assistants/associates toward the concept of developing an advanced practice postgraduate residency/fellowship program at a large academic medical center. Cureus.

[24] Ceyhun A.G., Deryal G.N., Sarıca H., Ungan M. (2025). Empowering medical residency training: A comparative analysis for understanding outpatient clinic demand and training needs. BMC Med. Educ..

[25] Nacht A., Martin J. (2020). An academic midwifery fellowship: Addressing a need for junior faculty development and interprofessional education. J. Midwifery Womens Health.

[26] Neller S., Beynon C., McLeskey N., Madden C., Edelman L.S. (2021). Development of a long-term care nurse residency program. J. Gerontol. Nurs..

[27] Rao A., Stamm C., Ihler E., Kruse D., Stone P. Program evaluation in women’s health integration for podiatric residents. Proceedings of the ACFAS Annual Scientific Meeting.

[28] Shofler D., He A., Lin T.L., Chuang C.T. (2019). An evaluation of off-service rotations in podiatric medicine and surgery residency training. J. Foot Ankle Surg..

[29] Testa E.J., Orman S., Bergen M.A., Ready L.V., Li N.Y., Gil J.A. (2022). Variability in hand surgery training among plastic and orthopaedic surgery residents. J. Am. Acad. Orthop. Surg. Glob. Res. Rev..

[30] Wang A.Y., Zhao H.C., Song Y.W., Xiong B.L., Guo Z.Y., Sun X.D., Pan Y.X., Sun W.D. (2025). Application of PBL in combination with the SP teaching method in the clinical teaching of orthopedics and traumatology. BMC Med. Educ..

[31] Wright J., Scardaville D. (2021). A nursing residency program: A window into clinical judgement and clinical decision making. Nurse Educ. Pract..

[32] Zgonis T., Hyer C.F. (2022). The legacy and impact of podiatric fellowship training. Clin. Podiatr. Med. Surg..

[33] Zhou E., Park B., Lin M.A., Driscoll A.M., DiFiori M., Rallis G., Fasulo S., Chu A., McGrath A. (2026). Gender biases in surgical residency and their association with postoperative outcomes: A qualitative study of residents in general and orthopedic surgery. Patient Educ. Couns..

[34] Page M.J., McKenzie J.E., Bossuyt P.M., Boutron I., Hoffmann T.C., Mulrow C.D., Shamseer L., Tetzlaff J.M., Akl E.A., Brennan S.E. (2021). The PRISMA 2020 statement: An updated guideline for reporting systematic reviews. BMJ.

[35] Carra M.C., Romandini P., Romandini M. (2025). Risk of bias evaluation of cross-sectional studies: Adaptation of the Newcastle-Ottawa Scale. J. Periodontal Res..

[36] (2011). Group OL of EW. The Oxford 2011 Levels of Evidence. Oxford Centre for Evidence-Based Medicine. https://www.cebm.ox.ac.uk/resources/levels-of-evidence/ocebm-levels-of-evidence.

[37] Schünemann H.J., Oxman A.D., Brozek J., Glasziou P., Jaeschke R., Vist G.E., Williams J.W., Kunz R., Craig J., Montori V.M. (2008). GRADE Working Group. Grading quality of evidence and strength of recommendations for diagnostic tests and strategies. BMJ.

